# Hybrid spiral-dynamic bacteria-chemotaxis algorithm with application to control two-wheeled machines

**DOI:** 10.1186/s40638-017-0059-1

**Published:** 2017-06-16

**Authors:** K. M. Goher, A. M. Almeshal, S. A. Agouri, A. N. K. Nasir, M. O. Tokhi, M. R. Alenezi, T. Al Zanki, S. O. Fadlallah

**Affiliations:** 1 Department of Informatics and Enabling Technologies, Lincoln University, Lincoln, New Zealand; 2 Department of Automatic Control and Systems Engineering, The University of Sheffield, Sheffield, UK; 3 Electronics Engineering Technology Department, College of Technological Studies, Public Authority for Applied Education and Training (PAAET), Adailiya, Kuwait; 4 Mechanical Engineering Department, Auckland University of Technology, Auckland, New Zealand

**Keywords:** Spiral dynamics, Bacteria chemotaxis, Two-wheeled inverted pendulum with new configuration, PD-like fuzzy logic control, Hybrid fuzzy logic control

## Abstract

This paper presents the implementation of the hybrid spiral-dynamic bacteria-chemotaxis (HSDBC) approach to control two different configurations of a two-wheeled vehicle. The HSDBC is a combination of bacterial chemotaxis used in bacterial forging algorithm (BFA) and the spiral-dynamic algorithm (SDA). BFA provides a good exploration strategy due to the chemotaxis approach. However, it endures an oscillation problem near the end of the search process when using a large step size. Conversely; for a small step size, it affords better exploitation and accuracy with slower convergence. SDA provides better stability when approaching an optimum point and has faster convergence speed. This may cause the search agents to get trapped into local optima which results in low accurate solution. HSDBC exploits the chemotactic strategy of BFA and fitness accuracy and convergence speed of SDA so as to overcome the problems associated with both the SDA and BFA algorithms alone. The HSDBC thus developed is evaluated in optimizing the performance and energy consumption of two highly nonlinear platforms, namely single and double inverted pendulum-like vehicles with an extended rod. Comparative results with BFA and SDA show that the proposed algorithm is able to result in better performance of the highly nonlinear systems.

## Background

Optimization algorithms play a dominant role in solving real problems [38, 58]. Bacterial foraging algorithm (BFA) [42] and spiral-dynamics algorithm (SDA) [50, 51] are well-known optimization techniques in solving real-world problems. Evolutionary algorithms (EA) have been used extensively in literature: soft computing techniques [46], particle swarm optimization [53, 55], incremental encoding [13], neural stochastic multi-scale optimization [9], multi-objective optimization [12, 23], multi-criteria optimization [43] and fuzzy logic and genetic programming [48].

Nasir et al. [33, 34, 36] proposed linear and nonlinear adaptive BFA where the bacteria step size is varied based on the combination of bacteria and iteration index. Chen and Lin [14], Farhat and El-Hawary [18] and Huang and Lin [22] utilized index and total number of chemotaxis to vary bacteria step size within a specified range. Niu et al. [39], Yan et al. [57] and Xu et al. [56] varied the step size within a user-defined range using combination of index and total number of iterations. Supriyono and Tokhi [49] developed various versions of BFA based on linear and nonlinear mathematical formulations to establish relationship between bacteria step size and their current fitness value. This relationship enables bacteria to have different step sizes in similar iteration as well as through the whole operation. There are other adaptive approaches considered the variation of the step size based on fitness value [16, 28, 29, 44, 45, 54]. Nasir et al. [30–32] proposed adaptive spiral-dynamic algorithm (ASDA) to establish relationship between spiral radius (*r*) and fitness value of each search point. They introduced schemes to make variation in the spiral radius within a specific range, enabling each search point to have different spiral radius in moving from one location to another location. Moreover, the movement step of each search agent was made with respect to its fitness value at the current location. As a result of the variation, there was improvement to the performance mainly on the accuracy of the final solution.

### Hybrid optimization techniques

Hybrid approach is the combination of two or more algorithms aimed to retain the advantages and eliminate the weaknesses of the original algorithms. This includes the synergization between different groups such as bio-inspired, nature-inspired, etc. Biswas et al. [10, 11] proposed hybrid BFA-PSO where a chemotactic strategy of bacteria was designed to represent exploitation part of the algorithm, while the exploration of optimum location was accomplished by PSO. The same approach using a constant step size was implemented by Korani [26], where the PSO operator was used to determine new direction of bacteria motion. Ghaffar et al. [19] adopted a modified PSO operator to determine new direction of bacteria to avoid local optima solution. Biswas et al. [11] proposed chemotactic differential evolution algorithm where adaptive chemotactic strategy of bacteria has been used to improve fitness accuracy of classical differential evolution (DE). Sinha et al. [47] implemented the same approach on an electric power system. Kim et al. [24] and Kim [25] used GA and BFA to tune a PID controller for automatic voltage regulation. Panigrahi and Ravikumar [40] and Hooshmand et al. [21] incorporated Nelder–Mead method into bacteria chemotaxis phase to enhance the search strategy and improve bacteria location. Other hybrid approaches involving BFA [41, 59] used bee colony algorithm and Tabu search.

### Limitations of BFA and SDA

BFA is a well-known bio-inspired algorithm. It has a comparable or better performance compared to other types of optimization algorithm [17]. Therefore, it has been adopted by many researchers worldwide to solve real-world problems in many areas [52]. However, BFA has a slow convergence speed and longer computation time. Due to this issue, the application of original BFA in online and offline tuning for solving a complex real-world problem is unsatisfactory [15]. On the other hand, SDA is a relatively new and a simple algorithm developed inspired from natural spiral phenomena on earth. It has a relatively fast convergence speed which can complement the drawback of BFA performance. Previous study showed that SDA has a similar or better performance compared to other differential evolutionary (DE) and particle swarm optimization (PSO) algorithms [50, 51]. However, SDA has a premature convergence issue where it hardly provides an optimal solution for complex problems.

### Hybrid spiral-dynamic bacteria-chemotaxis

A hybrid bacteria-chemotaxis spiral-dynamic algorithm (HSDBC) has been proposed by Nasir et al. [30–32] to synergize the chemotactic strategy of bacteria and ASDA. The chemotaxis phase in BFA was designed such that it represents exploration stage and placed at the first phase of the algorithm, while the ASDA as the exploitation stage and was placed at the second phase of the algorithm. The combination simplified the BFA algorithm and greatly reduced the total computation time of BFA. Moreover, comparison with original algorithms concluded that it improved the accuracy of the final solution and had the capability to avoid the local optima problem. HSDBC is a new variant of hybrid-type BFA-SDA algorithm developed to solve the issues aforementioned above. Our previous study showed that the algorithm outperformed both BFA and SDA algorithms in terms of accuracy in finding a global optima solution. Compared to BFA, the total computation time has been significantly reduced and its convergence speed has been considerably increased [31, 37].

**Fig. 1:**
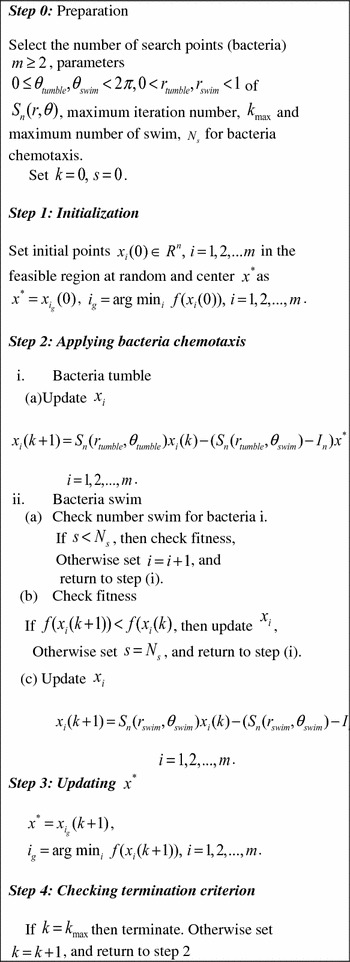
HSDBC algorithm for *n*-dimensional optimization

**Fig. 2:**
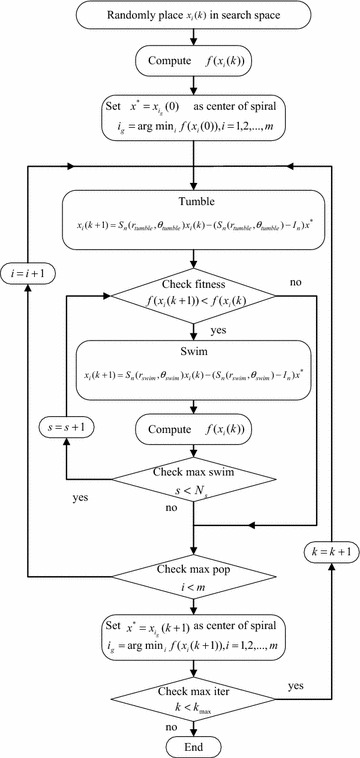
Flowchart of the HSDBC algorithm

Full description of the HSDBC algorithm for n-dimensional optimization is shown in Fig. 1. The description of the associated parameters used in the algorithm is shown in Table 1, and the corresponding flow chart is given in Fig. 2. The HSDBC algorithm has been tested to model and control nonlinear systems including flexible robot manipulator and a twin rotor system using a PD-like FLC [35, 37].

**Table 1:** HSDBC algorithm parameters

*θ*_tumble_	Bacteria angular displacement on *x*_*i*_ − *x*_*j*_ plane around the origin for tumbling
*θ*_swim_	Bacteria angular displacement on *x*_*i*_ − *x*_*j*_ plane around the origin for swimming
*r*_tumble_	Spiral radius from bacteria tumble
*r*_swim_	Spiral radius for bacteria swim
*m*	Number of search points
*k*_max_	Maximum iteration number
*N*_sw_	Maximum number of swim
*x*_*i*_ (*k*)	Bacteria position
*R* ^*n*^	*n* × *n* matrix

### Contribution overview and paper organization

Establishing the optimal control strategy for nonlinear dynamic systems, specifically inverted pendulum-based systems, has been and still remains a field of interest for a countless number of research studies due to their various promising real-life applications including personal transport systems and wheelchairs. This paper presents an extended study of the proposed algorithm in solving complex problem of two-wheeled inverted pendulum systems. We will implement HSDBC algorithm to control two different configurations of two-wheeled machines. A detailed simulation study of the HSDBC algorithm using several unimodal and multimodal benchmark functions can be found in the work of Nasir and Tokhi [37]. A hybrid fuzzy-like PD and I controller is designed and implemented on the two systems.

This paper is organized as follows: “Background” section introduces both ASDA and ABFA optimization algorithms, along with an explanation of the principle of HSDBC algorithm. In order to test and validate the proposed HSDBC algorithm on real dynamic systems, two case studies are considered in the study and are introduced in “Methods” section. “Case study I: single IP with an extended rod” section describes in details the first case study that involves a single inverted pendulum (IP) system. A double IP system with an extended rod is considered as the second case study and is presented in “Case study II: double IP with an extended rod” section. The results of the investigation are presented at the end of each of the previously mentioned sections, sections “Case study I: single IP with an extended rod” and “Case study II: double IP with an extended rod”. At last, the paper is concluded in “Conclusion” section.

## Methods

An inverted pendulum as a typical multi-input multi-output system has the characteristics of nonlinear, multivariable and close coupling Luo et al. [27]. The uniqueness and wide application of technology derived from this unstable system has drawn interest of many researchers including Akesson et al. [2], Askari et al. [5] and Balan et al. [6, 7]. There are various applications of IP configuration including design of walking gaits, wheelchairs, and personal transport systems.

The system considered in this paper is a two-wheeled machine (TWM) with an extendable rod as described by Goher et al. [20] and verified by Almeshal et al. [3, 4]. This system stabilizes it extendable intermediate body (IB) by controlling the wheel movements in a desired manner. A TWM is designed such that either the center of mass of the robot is above or below the axle joining two wheels. Statically unstable TWM have evoked a lot of interest in present decade [8]. Two case studies are used to test and validate the developed algorithm; single IP and double IP with an extended rod. For consistency, the two systems are considered to move along an inclined surface. The results of the simulation are shown in a comparative manner with three different optimization algorithms; BFA, SDA and HSDBC.

## Case study I: single IP with an extended rod

### System description

**Fig. 3:**
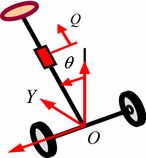
Two-wheeled vehicle with an extendable intermediate body

**Fig. 4:**
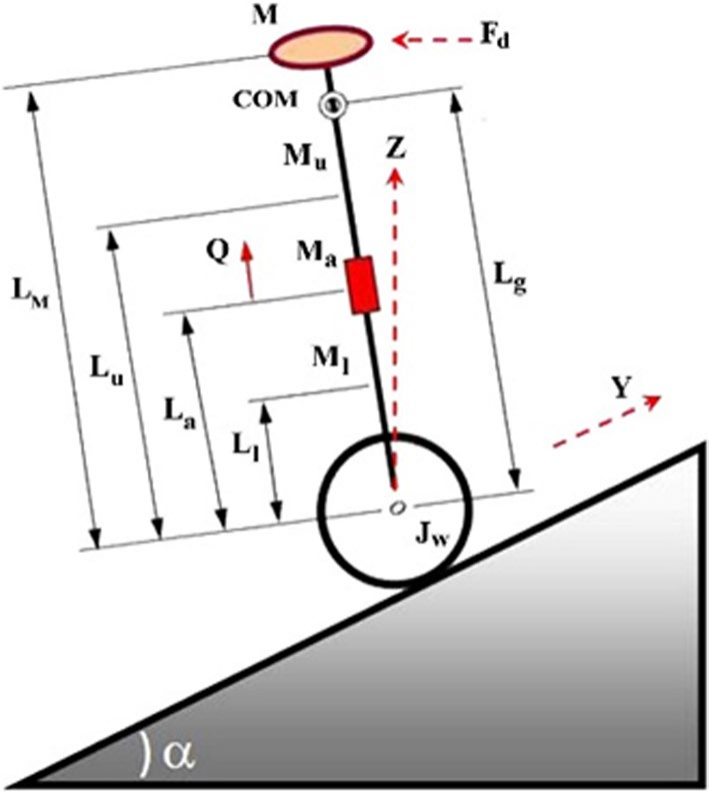
Schematic diagram of a single IP vehicle on an inclined plane

The system comprises a rod on an axle incorporating two wheels as shown in Figs. 3 and 4. The numerical parameters of the system are described in “Appendix 1”. Full details on the system description are available in Almeshal et al. [3, 4].

### Mathematical modeling of the single IP with an extended rod

Lagrange-Euler formulation is used to derive the system dynamic model using the following n-coordinates dynamic equations:

1 $$ \frac{\text{d}}{{{\text{d}}t}}\left( {\frac{\partial L}{{\partial \dot{q}_{i} }}} \right) - \frac{\partial L}{{\partial q_{i} }} = Q_{i} ,\quad i = 1,2, \ldots ,n $$

where $Q_{i}$ is generalized force vector and *q*_*i*_ is generalized coordinate vector. The coordinate vector is selected as:

2 $$ q_{i} = [\gamma \, \theta \, Q]^{\text{T}} $$

and the force vector as:

3 $$ Q_{i} = [F_{\text{c}} \, F_{\text{d}} \, F_{\text{a}} ]^{\text{T}} $$

The system equations of motion of the model can be written as:

4 $$ \frac{\text{d}}{{{\text{d}}t}}\left( {\frac{\partial L}{{\partial \dot{y}}}} \right) - \frac{\partial L}{\partial y} = F_{\text{c}} $$

5 $$ \frac{\text{d}}{{{\text{d}}t}}\left( {\frac{\partial L}{{\partial \dot{\theta }}}} \right) - \frac{\partial L}{\partial \theta } = F_{\text{d}} $$

6 $$ \frac{\text{d}}{{{\text{d}}t}}\left( {\frac{\partial L}{{\partial \dot{Q}}}} \right) - \frac{\partial L}{\partial Q} = F_{\text{a}} $$

Driving further the above equations yields the following nonlinear equations of motion of the system:

7 $$ \begin{aligned} & C_{7} \ddot{y} + (C_{15} + C_{16} Q)\ddot{\theta }\cos (\theta + \alpha ) \\ & \quad - (C_{15} + C_{16} Q)\dot{\theta }^{2} \sin (\theta + \alpha ) \\ & \quad + C_{16} \dot{Q}\dot{\theta }\cos (\theta + \alpha ) + C_{11} \sin \alpha = F_{c} \\ \end{aligned} $$

8 $$ \begin{aligned} & (C_{18} + QC_{16} )\ddot{y}\cos (\theta + \alpha ) \\ & \quad - (C_{18} + QC_{16} )\dot{y}\theta \sin (\theta + \alpha )C_{16} \dot{Q}\dot{y}\cos (\theta + \alpha ) \\ & \quad 2\ddot{\theta }(C_{12} Q^{2} + C_{13} Q + C_{14} ) + \dot{\theta }(4C_{12} Q + 2C_{13} \dot{Q}) \\ & \quad + \dot{y}\dot{\theta }^{2} \sin (\theta + \alpha )(C_{10} + M_{\text{u}} (C_{5} + Q) + M_{\text{m}} (C_{6} + Q)) \\ & \quad - \dot{\theta }\sin \theta (C_{10} g + M_{\text{u}} g(C_{5} + Q) + M_{\text{m}} g(C_{6} + Q)) = F_{\text{d}} \\ \end{aligned} $$

9 $$ \begin{aligned} & 2C_{8} \ddot{Q} - C_{16} \dot{\theta }\dot{y}\cos (\theta + \alpha ) - 2C_{12} Q\dot{\theta }^{2} \\ & \quad - C_{13} \dot{\theta }^{2} - C_{16} g\cos \theta = F_{\text{a}} \\ \end{aligned} $$

Detailed explanations of the constant parameters appearing in Eqs. (4)–(9) are formulated in “Appendix 2”.

### Control strategy

**Fig. 5:**
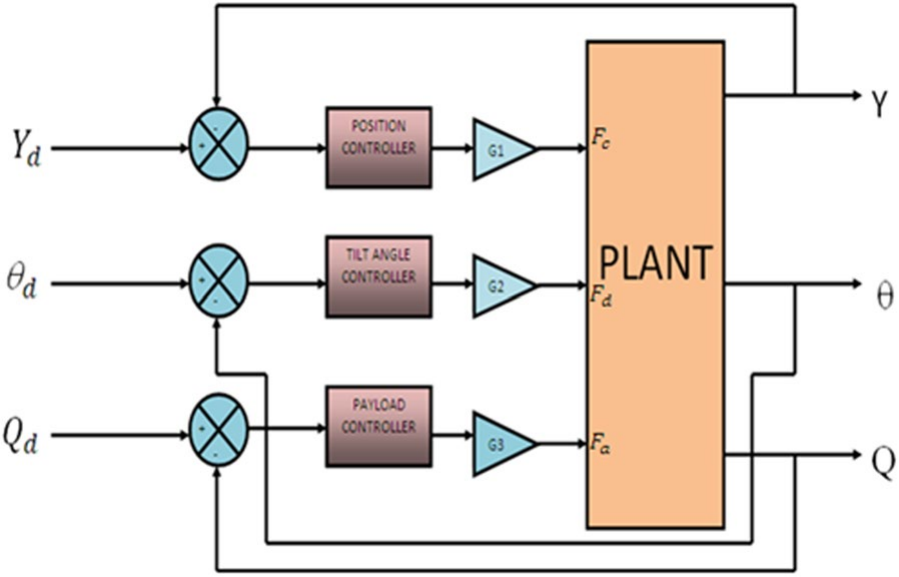
System block diagram

**Fig. 6:**
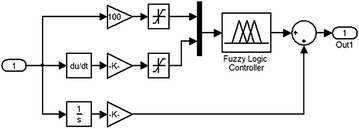
Fuzzy PD + I controller

Three independent control loops, shown in Fig. 5, are implemented on the system. Fuzzy PD-like combined with conventional integrator is designed as shown in Fig. 6. The three control loops are working to: stabilize the IB at the vertical upright position, keep the cart wheels within a specified linear position from a predefined reference while moving on an inclined surface, and to control the linear displacement of the payload along the IB.

The inputs to the three control loops are the error signal, change of error and the sum of previous errors. The system inputs are the driving force $F_{\text{c}}$, the linear actuator force $F_{\text{a}}$ and the disturbance force $F_{\text{d}}$. FLC controllers are developed based on Mamdani-type fuzzy inference engine with (25) fuzzy rules shown in Table 2.

**Table 2:** Fuzzy rule base

*ê*	*e*				
	NB	NS	Z	PS	PB
NB	NB	NB	NB	NS	Z
NS	NB	NB	NS	Z	PS
Z	NB	NS	Z	PS	PB
PS	NS	Z	PS	PB	PB
PB	Z	PS	PB	PB	PB

### Constrained optimization

The optimization process is constrained within the stability region of the system. Each parameter has a feasible interval that guarantees the stability of the system within the defined gain limits. Table 3 presents the limits of each parameter which represent the search space of each of the three addressed algorithms. Those parameters were obtained through a manual tuning exercise of the system.

**Table 3:** Boundary limits of the controller gain parameters

Gain parameters	Minimum value	Maximum value
*Kp*_*1*_	4	5
*Kd*_*1*_	3	4
*Ki*_*1*_	0.4	0.8
*Kp*_*2*_	4	5
*Kd*_*2*_	3	4
*Ki*_*2*_	1	1.3
*Kp*_*3*_	10	13
*Kd*_*3*_	15	20
*Ki*_*3*_	2	3

#### Objective functions

The performance index of the system is chosen as the minimum mean squared error (MSE) of each control loop. The MSE is calculated for each control loop of the vehicle system using the following equations:

10 $$ \begin{aligned} {\text{Objective}}\_{\text{Function1}} & = \hbox{min} \left[ {\frac{1}{N}\sum\limits_{i = 1}^{N} {(Y_{\text{d}} - Y_{\text{m}} )^{2} } } \right] \\ {\text{Objective}}\_{\text{Function2}} & = \hbox{min} \left[ {\frac{1}{N}\sum\limits_{i = 1}^{N} {(\theta_{\text{d}} - \theta_{\text{m}} )^{2} } } \right] \\ {\text{Objective}}\_{\text{Function3}} & = \hbox{min} \left[ {\frac{1}{N}\sum\limits_{i = 1}^{N} {(Q_{\text{d}} - Q_{\text{m}} )^{2} } } \right] \\ \end{aligned} $$

The objective function of the system is calculated based on the total MSE which can be expressed as:

11 $$ J = \sum\limits_{i = 1}^{3} {{\text{Objective}}\_{\text{function}}(i\text{)}} $$

The parameters used to implement the three optimization algorithms are shown in Tables 4, 5 and 6 and the calculated optimized parameters are shown in Table 7. The data shown in Table 8 gives the minimum cost functions due to the implementation of the three optimization algorithms where the HSDBC algorithm was able to give the minimum cost function compared to the BFA and SDA optimizations.

**Table 4:** BFA parameters

P	S	Nc	Ns	Nre	Ned	Ped	Sr
9	40	10	6	2	2	0.25	S/2

**Table 5:** SDA parameters

P	R	Theta	Initial points	Iterations
9	0.9	*π*/4	5	90

**Table 6:** HSDBC parameters

P	R	Rzw	Ns	Theta	Initial points	Iterations
9	0.95	0.55	2	*π*/4	5	90

**Table 7:** Optimized gain values

	Parameter	BFA	SDA	HSDBC
Loop 1	*Kp*_*1*_	4.2287	4.0000	4.0003
	*Kd*_*1*_	3.0064	3.1065	3.0089
	*Ki*_*1*_	0.7380	0.6773	0.7267
Loop 2	*Kp*_*2*_	4.5638	4.3183	4.7770
	*Kd*_*2*_	3.2615	3.6085	3.4461
	*Ki*_*2*_	1.0322	1.2380	1.1306
Loop 3	*Kp*_*3*_	11.4488	10.7368	11.3992
	*Kd*_*3*_	19.3417	17.1030	18.0021
	*Ki*_*3*_	2.6508	2.0113	2.6529

**Table 8:** Cost functions

Minimum cost function value	BFA	SDA	HSDBC
*J*	0.922	0.8804	0.8517

### Simulation results

**Fig. 7:**
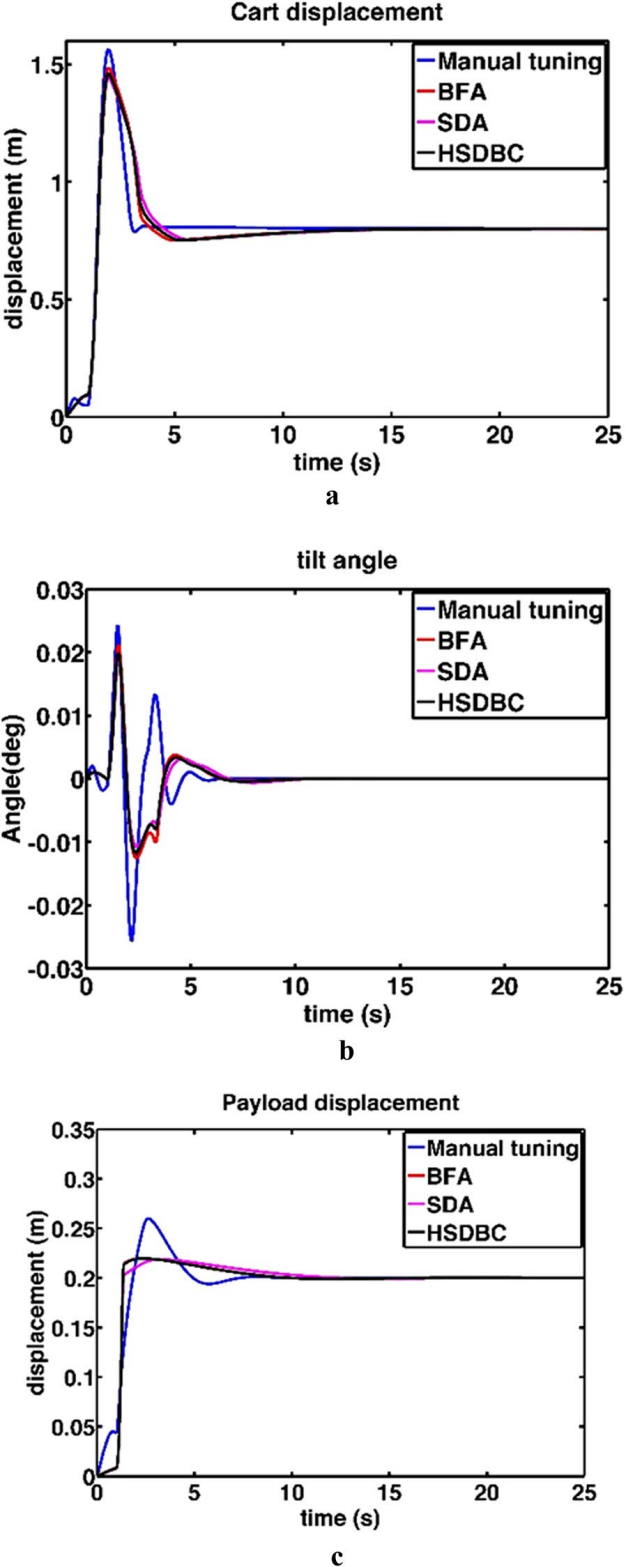
Performance of the single IP. **a** Linear displacement of the cart, **b** tilt angle of the intermediate body, **c** linear displacement of the payload

**Fig. 8:**
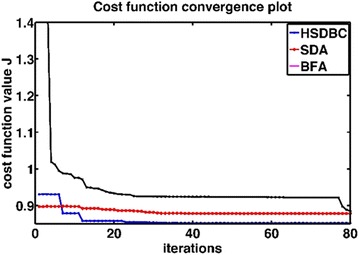
Cost function convergence

Four consecutive simulation runs of the system model yielded the performance of the system as shown in Fig. 7. As noted from Fig. 6; the three optimization algorithms; BFA, SDA and HSDBC, resulted generally in a satisfactory performance of the system. However, HSDBC algorithm showed a superior performance in minimizing the percentage overshoot in the payload displacement as appeared in Fig. 7c. As per the tilt angle shown in Fig. 7b, all the three algorithms behaved the same in terms of minimizing the level and period of oscillations. As per convergence graph shown in Fig. 8 show that the three algorithms resulted in similar convergence of the cost function within around 25 iterations. However, the HSDBC was faster in convergence of the cost function too early if compared to the BFA and SDA algorithms.

**Fig. 9:**
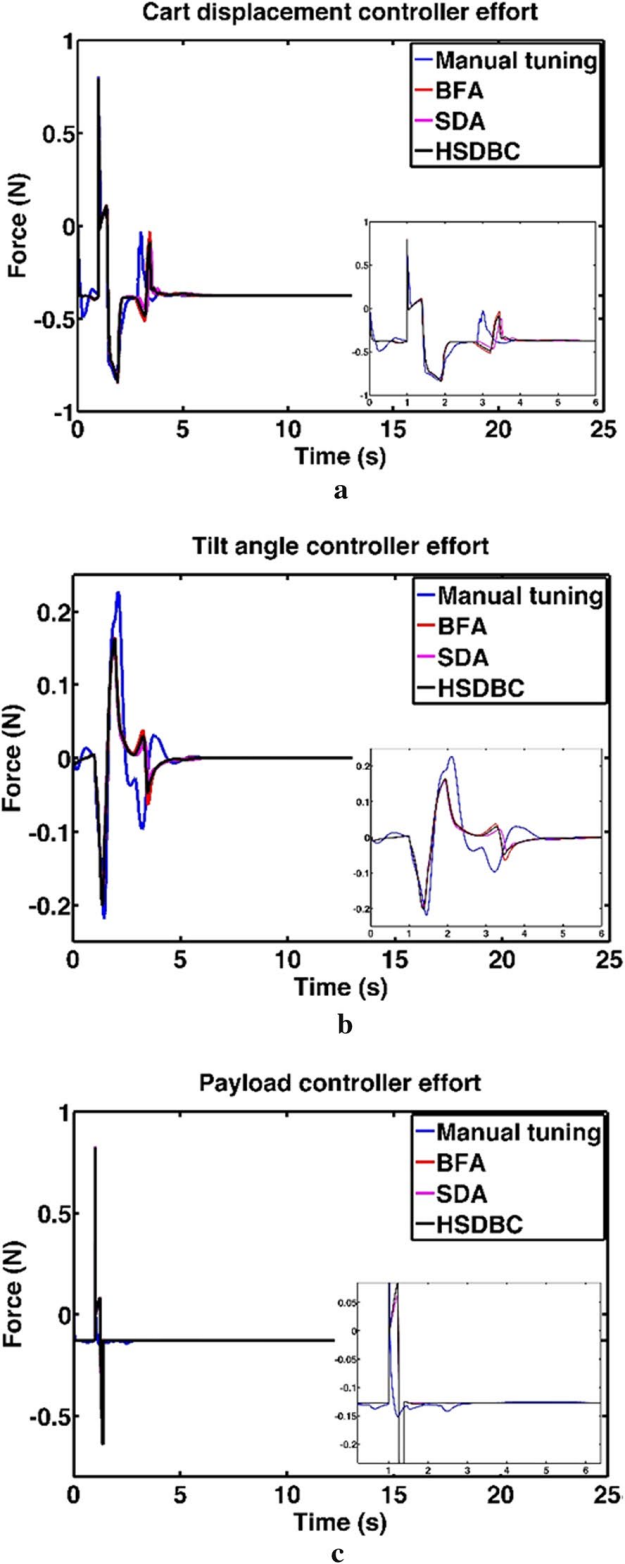
Control effort components in a single IP. **a** Cart control effort, **b** tilt angle control effort, **c** linear actuator driving force

Attention has been focused on energy consumption in this investigation. The control effort components as a measure of energy consumption are shown in Fig. 9. It is noted that the control effort required in the transient range; the three algorithms yielded nearly close results. However, the HSDC was more robust as it resulted less oscillation of the control effort components in the magnified areas of the plots. Significant amount of energy saving has been achieved specifically in the cart and tilt angle control efforts as appeared in Fig. 9a, b. Furthermore, the HSDBC resulted in a great improvement in the control effort for the payload; this can be demonstrated by the significant improvement shown in Fig. 9c in terms of less oscillations and the short time taken by the control signal to stabilize.

## Case study II: double IP with an extended rod

**Fig. 10:**
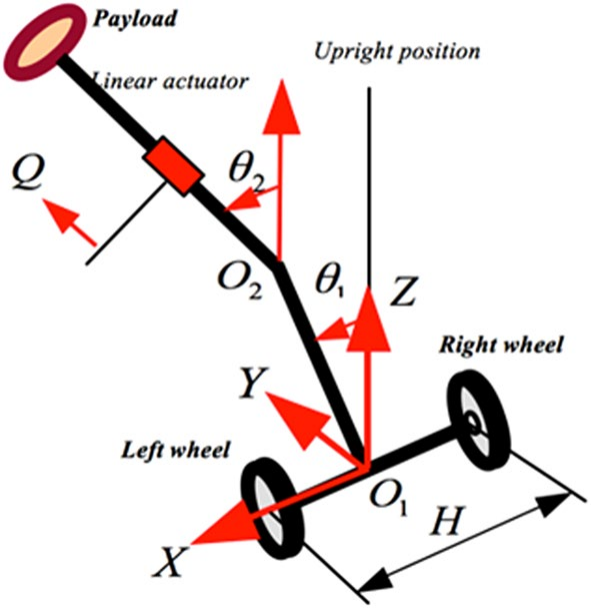
Axonometric diagram of a double IP vehicle

In this case study, an additional link is added and hence increasing the degrees of the freedom (DOF) and the complexity of the structure. The double IP with such configuration shown in Fig. 10 is mimicking the scenario of a wheelchair on only two wheels which has been studied significantly by Ahmad and Tokhi [1].

The design of the two-wheeled robotic vehicle is based on double inverted pendulum system with a movable payload moving on an inclined surface with five DOF. The increased DOFs will enable the vehicle to maneuver freely in all directions and in different environments. Moreover, the second link provides an extended height to lift up the payload to a demanded height. The system equations of motion are presented with five highly coupled differential equations as follows:

12 $$ \begin{aligned} & 2{\kern 1pt} C_{27} \ddot{\delta }_{L} + 2C_{1} \ddot{\delta }_{\text{R}} + C_{6} \ddot{\theta }_{1} \cos (\theta_{1} + \alpha ) \\ & \quad - C_{6} \dot{\theta }_{1}^{2} \sin (\theta_{1} + \alpha ) \\ & \quad + 0.5(C_{25} + C_{26} Q) \\ & \quad \left( {\ddot{\theta }_{2} \cos (\theta_{2} + \alpha ){\kern 1pt} - {\kern 1pt} \dot{\theta }_{2}^{2} \sin (\theta_{2} + \alpha )} \right) \\ & \quad + 0.5C_{28} \dot{Q}\dot{\theta }_{2} \cos (\theta_{2} + \alpha ) \\ & \quad + C_{16} g\sin \alpha = T_{L} - T_{fL} \\ \end{aligned} $$

13 $$ \begin{aligned} & 2{\kern 1pt} C_{27} \ddot{\delta }_{\text{R}} + 2C_{1} \ddot{\delta }_{\text{L}} + C_{6} \ddot{\theta }_{1} \cos (\theta_{1} + \alpha ) \\ & \quad - C_{6} \dot{\theta }_{1}^{2} \sin (\theta_{1} + \alpha ) \\ & \quad + 0.5(C_{25} + C_{26} Q) \\ & \quad \left( {\ddot{\theta }_{2} \cos (\theta_{2} + \alpha ){\kern 1pt} - {\kern 1pt} \dot{\theta }_{2}^{2} \sin (\theta_{2} + \alpha )} \right) \\ & \quad + 0.5C_{28} \dot{Q}\dot{\theta }_{2} \cos (\theta_{2} + \alpha ) \\ & \quad + C_{16} g\sin \alpha = T_{\text{R}} - T_{\text{fR}} \\ \end{aligned} $$

14 $$ \begin{aligned} & 2{\kern 1pt} C_{2} \ddot{\theta }_{1} + \left( {C_{5} + M_{{2{\text{u}}}} {\kern 1pt} L_{1} (C_{8} + Q) + M{\kern 1pt} L_{1} (C_{9} + Q)} \right) \\ & \quad \left( {\ddot{\theta }_{2} \cos (\theta_{1} - \theta_{2} ){\kern 1pt} - \dot{\theta }_{2} (\dot{\theta }_{1} - \dot{\theta }_{2} )\sin (\theta_{1} - \theta_{2} )} \right) \\ & \quad + \dot{\theta }_{2} \cos (\theta_{1} - \theta_{2} )\left( {\dot{Q}(M_{{2{\text{u}}}} L_{1} + M{\kern 1pt} L_{1} )} \right) \\ & \quad + C_{6} (\ddot{\delta }_{\text{L}} + \ddot{\delta }_{\text{R}} )\cos (\theta_{1} + \alpha ) \\ & \quad + C_{6} (\dot{\delta }_{\text{L}} + \dot{\delta }_{\text{R}} )\sin (\theta_{1} + \alpha )(\dot{\theta }_{1}^{2} - \dot{\theta }_{1} ) \\ & \quad + \dot{\theta }_{1}^{2} \dot{\theta }_{2} \sin (\theta_{1} - \theta_{2} ) \\ & \quad \left( {C_{5} + M_{{2{\text{u}}}} {\kern 1pt} L_{1} (C_{8} + Q) + ML_{1} (C_{9} + Q)} \right) \\ & \quad - g{\kern 1pt} C_{14} \dot{\theta }_{1} \sin \theta_{1} = 0.5(T_{\text{R}} + T_{\text{L}} ) \\ \end{aligned} $$

15 $$ \begin{aligned} & C_{19} \ddot{Q} - {\kern 1pt} {\kern 1pt} 0.5\dot{\theta }_{2}^{2} (2C_{19} Q + C_{22} ) \\ & \quad - C_{23} \dot{\theta }_{1} \dot{\theta }_{2} \cos (\theta_{1} - \theta_{2} ) \\ & \quad - 0.5C_{25} \dot{\theta }_{2} (\dot{\delta }_{\text{L}} + \dot{\delta }_{\text{R}} )\cos (\theta_{2} + \alpha ) \\ & \quad + g{\kern 1pt} C_{18} \cos \theta_{2} = F_{\text{a}} - F_{\text{fa}} \\ \end{aligned} $$

16 $$ \begin{aligned} & \ddot{\theta }_{2} (C_{19} {\kern 1pt} Q^{2} + C_{20} {\kern 1pt} Q + C_{21} ) + \dot{\theta }_{2} (2C_{19} {\kern 1pt} Q^{2} + C_{22} ) \\ & \quad + \ddot{\theta }_{1} \cos (\theta_{1} - \theta_{2} )(C_{23} {\kern 1pt} Q + C_{24} ) \\ & \quad - \dot{\theta }_{1} (\dot{\theta }_{1} - \dot{\theta }_{2} )\sin (\theta_{1} - \theta_{2} )(C_{23} {\kern 1pt} Q + C_{24} ) \\ & \quad + C_{23} \dot{\theta }_{1} \cos (\theta_{1} - \theta_{2} ) \\ & \quad + 0.5{\kern 1pt} (\ddot{\delta }_{\text{L}} + \ddot{\delta }_{\text{R}} )\cos (\theta_{2} + \alpha )(C_{25} {\kern 1pt} Q + C_{26} ) \\ & \quad - 0.5{\kern 1pt} (\dot{\delta }_{\text{L}} + \dot{\delta }_{\text{R}} )\dot{\theta }_{2} \sin (\theta_{2} + \alpha )(C_{25} {\kern 1pt} Q + C_{26} ) \\ & \quad + 0.5{\kern 1pt} C_{25} (\dot{\delta }_{\text{L}} + \dot{\delta }_{\text{R}} )\cos (\theta_{2} + \alpha ) \\ & \quad - \dot{\theta }_{1} {\kern 1pt} {\kern 1pt} \dot{\theta }_{2} \sin (\theta_{1} - \theta_{2} )(C_{23} {\kern 1pt} Q + C_{24} ) \\ & \quad + 0.5{\kern 1pt} {\kern 1pt} \dot{\theta }_{2}^{2} (\dot{\delta }_{\text{L}} + \dot{\delta }_{\text{R}} )\sin (\theta_{2} + \alpha )(C_{25} {\kern 1pt} Q + C_{26} ) \\ & \quad - g{\kern 1pt} \dot{\theta }_{2} \sin \theta_{2} (C_{17} + C_{18} {\kern 1pt} Q) = T_{\text{M}} - T_{\text{fM}} - L_{\text{d}} F_{\text{d}} \\ \end{aligned} $$

### Control strategy

**Fig. 11:**
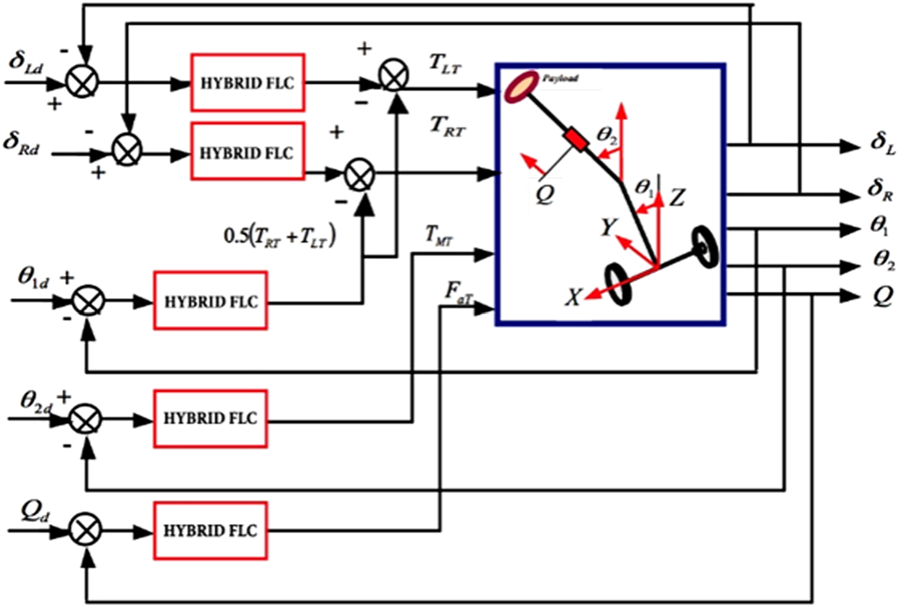
Block diagram of the vehicle control system

A robust hybrid fuzzy logic control strategy (FLC) with five control loops is developed. The control strategy block diagram is presented in Fig. 11, to control the vehicle and to counteract the disturbances occurring due to different movement scenarios.

**Fig. 12:**
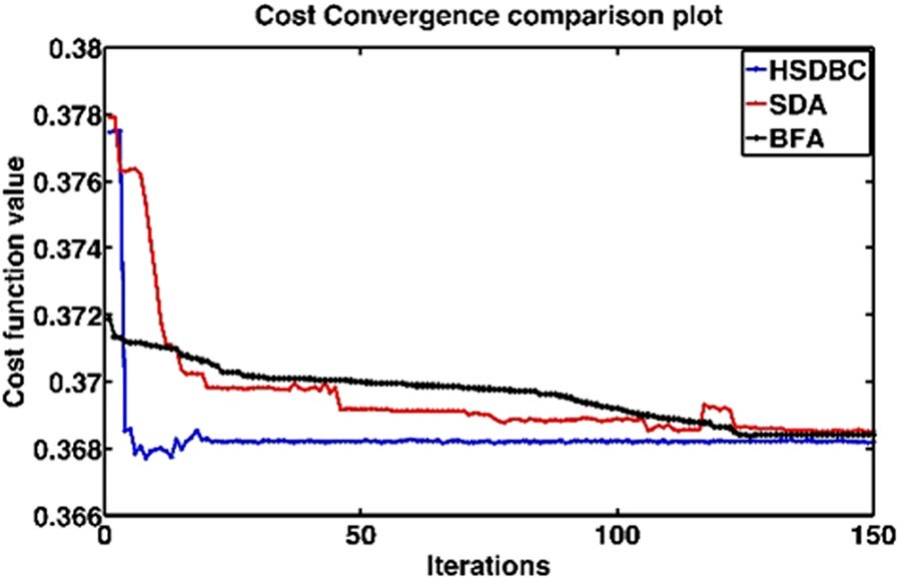
Cost function convergence plot for BFA, SDA and HSDBC algorithms

The control system of the vehicle consists of five hybrid FLC controllers with a total of 15 gain parameters. The gain parameters were first tuned heuristically in order to test the controller as well as to find the boundaries of the search space of those gain parameters. The same optimization algorithms, SDA, BFA and HSDBC, are implemented in order to optimize the vehicle control system parameters. The performance index of the system is chosen as the minimum mean squared error (MSE) for each control loop and defined as:

17 $$ \begin{aligned} {\text{MSE}}\,1 = \hbox{min} \left\{ {\frac{1}{N}\sum\limits_{i = 1}^{N} {(\delta_{\text{Ld}} - \delta_{\text{Lm}} )^{2} } } \right\} \hfill \\ {\text{MSE}}\;2 = \hbox{min} \left\{ {\frac{1}{N}\sum\limits_{i = 1}^{N} {(\delta_{\text{Rd}} - \delta_{\text{Rm}} )^{2} } } \right\} \hfill \\ {\text{MSE}}\;3 = \hbox{min} \left\{ {\frac{1}{N}\sum\limits_{i = 1}^{N} {(\theta_{{1{\text{d}}}} - \theta_{{1{\text{m}}}} )^{2} } } \right\} \hfill \\ {\text{MSE}}\;4 = \hbox{min} \left\{ {\frac{1}{N}\sum\limits_{i = 1}^{N} {(\theta_{{2{\text{d}}}} - \theta_{{2{\text{m}}}} )^{2} } } \right\} \hfill \\ {\text{MSE}}\;5 = \hbox{min} \left\{ {\frac{1}{N}\sum\limits_{i = 1}^{N} {(Q_{\text{d}} - Q_{\text{m}} )^{2} } } \right\} \hfill \\ \end{aligned} $$

The objective function is chosen as the summation of the MSE of the system expressed as:

18 $$ J = {\text{MSE}}_{1} + {\text{MSE}}_{2} + {\text{MSE}}_{3} + {\text{MSE}}_{4} + {\text{MSE}}_{5} $$

Minimization of the objective function *J* is used to find the optimal controller gain parameters that result in the minimum control loop errors in the stability region of the system.

### Constrained optimization

With the complexity of the model, slight changes in the control gain parameters will result in oscillations in the system response and may lead to instability of the vehicle. Constrained optimization techniques are used to avoid this problem occurring while optimizing the control system parameters. The optimization process is constrained within the stability region of the system. This is achieved by defining a feasible interval for each control parameter shown in Table 9, which assures the stability of the system.

**Table 9:** Boundary limits of the controller gain parameters

	Parameter	Lower	Upper
Loop 1	*Kp*_*1*_	1.5	2.4
	*Kd*_*1*_	0.5	1
	*Ki*_*1*_	0.9	1.4
Loop 2	*Kp*_*2*_	5	6.5
	*Kd*_*2*_	2.5	4
	*Ki*_*2*_	1.5	2
Loop 3	*Kp*_*3*_	8	12
	*Kd*_*3*_	7.5	9
	*Ki*_*3*_	0	0.5
Loop 4	*Kp*_*4*_	8	10
	*Kd*_*4*_	5	8
	*Ki*_*4*_	0	0.5
Loop 5	*Kp*_*5*_	30	50
	*Kd*_*5*_	10	20
	*Ki*_*5*_	1	10

### Results and discussion

This simulation scenario allows comparing the performance of the HSDBC with other similar optimization algorithms. Tables 10, 11 and 12 provide the simulation parameters used for BFA, SDA and HSDBC algorithms, respectively. The optimized control gain parameters reported by each optimization algorithm are presented in Table 13, whereas Table 14 provides the minimum cost function calculated by each of the optimization algorithms. Clearly, the HSDBC algorithm has found the minimum cost function value of 0.3682.

**Table 10:** BFA parameters

P	S	Nc	Ns	Nre	Ned	Ped	Sr
15	20	14	6	2	2	0.25	S/2

**Table 11:** SDA parameters

P	R	Theta	Initial points	Iterations
15	0.95	*π*/4	10	150

**Table 12:** HSDBC parameters

P	R	Rzw	Ns	Theta	Initial points	Iterations
15	0.95	0.55	2	*π*/4	10	150

**Table 13:** Optimized gain values

	Parameter	BFA	SDA	HSDBC
Loop 1	*Kp*_*1*_	2.0729	2.3452	2.1566
	*Kd*_*1*_	0.8572	0.8714	0.8095
	*Ki*_*1*_	1.3925	1.2778	1.2026
Loop 2	*Kp*_*2*_	6.0155	5.1504	5.1530
	*Kd*_*2*_	2.8185	3.1264	2.6917
	*Ki*_*2*_	1.6390	1.9794	1.8754
Loop 3	*Kp*_*3*_	8.7514	11.3330	11.4514
	*Kd*_*3*_	8.1889	8.3229	8.9946
	*Ki*_*3*_	0.2449	0.2731	0.3771
Loop 4	*Kp*_*4*_	8.9718	9.8522	9.9903
	*Kd*_*4*_	5.1315	6.7829	6.6239
	*Ki*_*4*_	0.0071	0.0532	0.0410
Loop 5	*Kp*_*5*_	49.9646	36.5230	36.6753
	*Kd*_*5*_	13.6834	14.2519	14.3583
	*Ki*_*5*_	4.0408	5.3567	5.4203

**Table 14:** Cost functions

Minimum cost function	BFA	SDA	HSDBC
*J*	0.3684	0.3685	0.3682

**Fig. 13:**
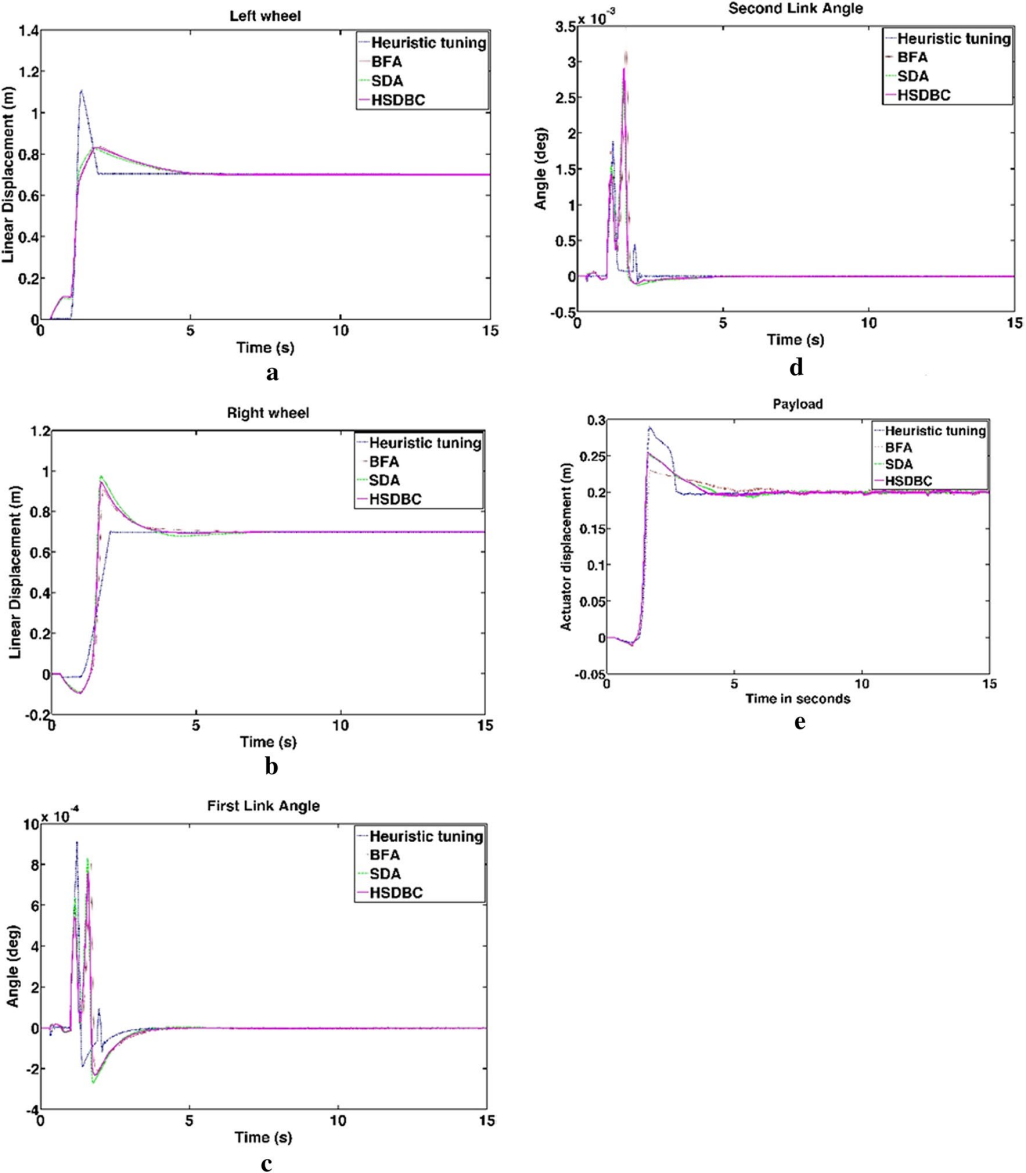
Performance of the double IP. **a** The linear displacement of the left wheel, **b** the linear displacement of the right wheel, **c** the tilt angle of the first link, **d** the tilt angle of the second link, **e** the payload linear actuator displacement

**Fig. 14:**
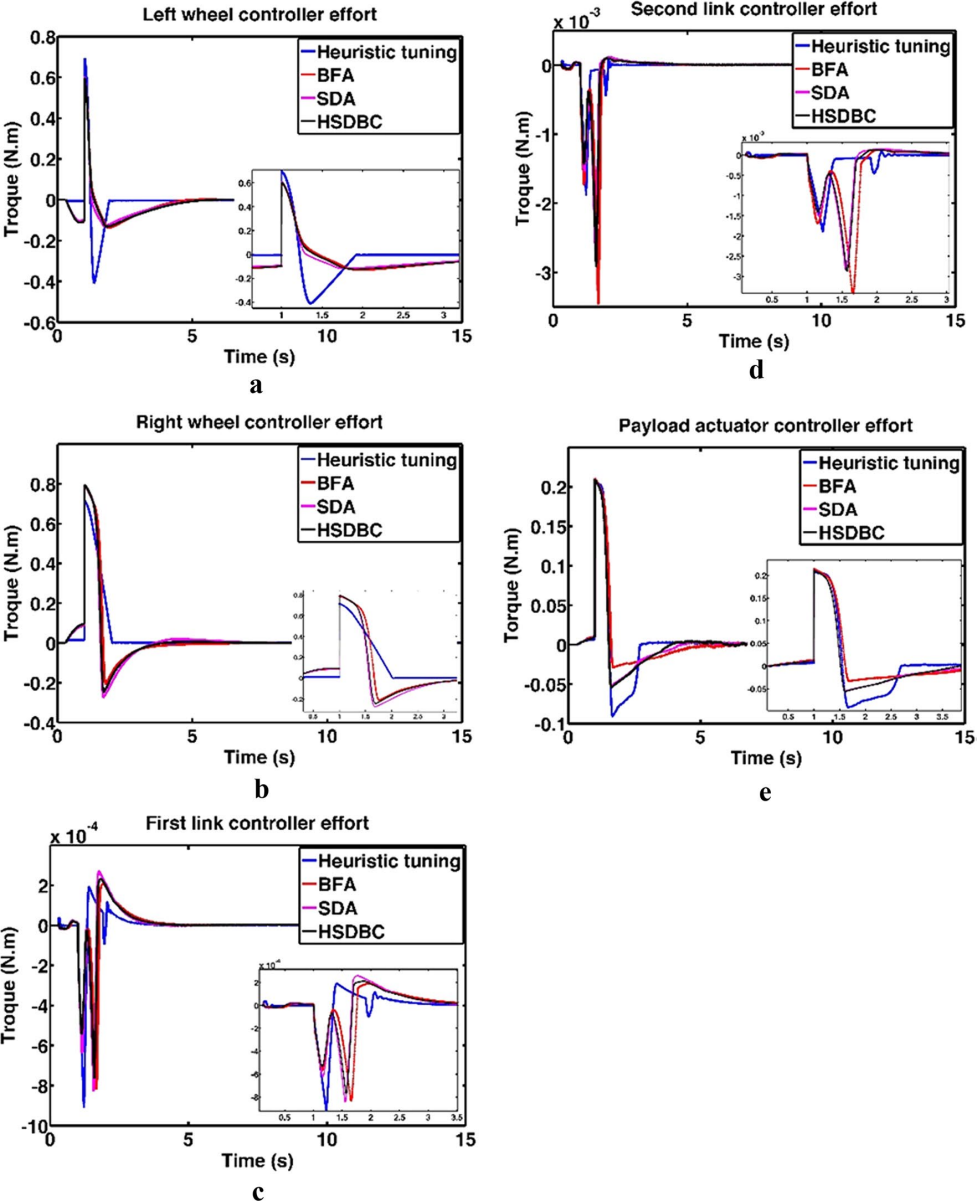
Control effort components in double IP case. **a** Left wheel torque, **b** right wheel torque, **c** first link torque, **d** second link torque, **e** linear actuator driving force

Figure 13 shows the system response based on the optimized control parameters obtained by the implementation of the BFA, SDA and HSDBC algorithms in comparison to the manual-tuned gain parameters. It can be noted that BFA, SDA and HSDBC are of much similar effect on the system response by finding stable solutions, lowering the overshoots and improved steady-state error. However, HSDBC algorithm has a superior performance in minimizing the percentage overshoot and the settling time for the linear displacement of the left and right wheel as shown in Fig. 13a, b and the tilt angles of the two pendula as shown in Fig. 13c, d. Furthermore, HSDBC-optimized gain parameters clearly improved the settling time of the payload actuator displacement as depicted in Fig. 13e. As can be noticed from the cost function convergence plots shown in Fig. 12, the HSDBC algorithm cost function has converged into the minimum value within approximately 25 iterations. However, the BFA and SDA algorithms seem to need more iterations to settle into their best-found minimum values presented in Table 14. HSDBC has successfully found the minimum cost function and proved its speed in convergence. In terms of the control output components shown in Fig. 14, the control efforts was minimized by the implementation of HSDBC algorithm for the left wheel, first link and the payload linear actuator. However, the heuristic tuning yields better results in case of the right wheel and the second link. This seems to be accompanied with a poor response of the system, in terms of increased disturbance period and higher gain values, if compared to the results obtained by the HSDBC algorithm.

## Conclusions

A novel hybrid spiral-dynamics bacteria-chemotaxis (HSDBC) optimization algorithm has been proposed. Chemotactic strategy of bacteria through spiral tumble and swim actions of bacteria is adopted to improve exploration strategy of SDA. Moreover, spiral radius and angular displacement of spiral model is made adaptive to enhance the movement of bacteria within feasible region. Incorporating these two schemes have successfully saved the SDA from getting trapped into local optima point and provides faster convergence. The proposed algorithm has been utilized to optimize the performance of two different IP platforms; single and double IP with a new configuration of an extended intermediate body. Simulation results have shown that the proposed hybrid algorithm outperformed its predecessor algorithms (BFA and SDA) in terms of increased convergence speed and better fitness accuracy. Furthermore, implementation of the HSDBC yielded significant saving in the energy consumption of the two tested platforms.

Future work will consider investigating standard PID tuning methods, such as Ziegler–Nichols method, and evaluating and comparing their performance with the HSDBC algorithm.

## Appendices

### Appendix 1: Single IP parameters

**Table:**

	Description	Unit
$L_{\text{P}}$	Position of the COM of the payload	m
$L_{\text{u}}$	Position of the COM of the rod’s upper part	m
$L_{\text{a}}$	Position of COM of the linear actuator	m
$L_{\text{l}}$	Position of COM of the lower part of the rod	m
*Q*	Displacement of the linear actuator	m
*Y*	Linear displacement of the vehicle	m
*θ*	The angular displacement of the IB	rad
$\dot{\gamma }$	Linear velocity of the IB	m/s
$\dot{\theta }$	Angular velocity of the IB	rad/s
$\ddot{\gamma }$	Linear acceleration of the IB	m/s^2^
$\ddot{\theta }$	Angular acceleration	rad/s^2^
$\ddot{Q}$	Linear acceleration of the attached payload	m/s^2^
$M_{\text{L}}$	Mass of the lower part of the rod	kg
$M_{\text{a}}$	Mass of the linear actuator	kg
$M_{\text{u}}$	Mass of the upper part of the rod	kg
$M$	Payload mass	kg
$M_{\text{W}}$	Wheel mass	kg
$M_{c}$	Cart mass	kg
$R_{\text{W}}$	Radius of the wheel	m
*g*	Gravitational acceleration	m/s^2^
$T_{\text{c}}$	Kinetic energy of the cart	N m
$T_{L}$	Kinetic energy of the lower part of the rod	N m
$T_{\text{a}}$	Kinetic energy of the linear actuator	N m
$T_{\text{u}}$	Kinetic energy of the upper part of the rod	N m
$T_{p}$	Kinetic energy of the payload	N m
$V_{\text{L}}$	Potential energy of the lower part of the rod	N m
$V_{\text{a}}$	Potential energy of the linear actuator	N m
$V_{\text{u}}$	Potential energy of the upper part of the rod	N m
$V_{\text{p}}$	Potential energy of the payload	N m
$V_{\text{c}}$	Potential energy of the cart	N m
*α*	Inclination angle	rad

### Appendix 2: Single IP constants

$$ \begin{aligned} C_{1} & = 4L_{\rm l} + L_{\rm l}^{2} + 4L_{\rm l} L_{\rm u} \\ C_{2} & = 4L_{\rm l} + 2L_{\rm u} \\ C_{3} & = 4L_{\rm l}^{2} + 4L_{\rm u}^{2} + 8L_{\rm l} L_{\rm u} \\ C_{4} & = 4L_{\rm l} + 4L_{\rm u} \\ C_{5} & = 2L_{\rm l} + L_{\rm u} \\ C_{6} & = 2L_{\rm l} + 2L_{\rm u} \\ C_{7} & = M_{\rm c} + M_{\rm l} + M_{\rm a} + M_{\rm u} + M_{\rm m} \\ C_{8} & = M_{\rm c} + M_{\rm l} + M_{\rm a} + M_{\rm u} + M_{\rm m} \\ C_{9} & = \frac{1}{2}M_{\rm l} L_{\rm l}^{2} + \frac{1}{2}J_{\rm l} + \frac{1}{2}M_{\rm a} L_{\rm a}^{2} + \frac{1}{2}J_{\rm a} \\ C_{10} & = M_{\rm l} L_{\rm l} + M_{\rm a} L_{\rm a} \\ C_{11} & = (M_{\rm c} + M_{\rm l} + M_{\rm a} + M_{\rm u} + M_{\rm m} )g \\ C_{12} & = \frac{1}{2}M_{\rm u} + \frac{1}{2}M_{\rm m} + \frac{1}{2}M_{\rm u} + \frac{1}{2}M_{\rm m} \\ C_{13} & = \frac{1}{2}(C_{2} + C_{4} )M_{\rm u} + \frac{1}{2}M_{\rm u} (C_{2} - 2L_{\rm g} ) + \frac{1}{2}M_{\rm m} (C_{4} - 2L_{\rm g} ) \\ C_{14} & = C_{9} + \frac{1}{2}C_{1} M_{\rm u} + C_{3} M_{\rm m} + \frac{1}{2}(M_{\rm u} + M_{\rm m} )L_{\rm g}^{2} - C_{5} M_{\rm u} L_{\rm g} + \frac{1}{2}C_{1} M_{\rm u} \\ & \quad + \frac{1}{2}M_{\rm m} L_{\rm g}^{2} - C_{6} M_{\rm m} L_{\rm g} \\ C_{15} & = C_{10} + C_{5} M_{\rm u} + C_{6} M_{\rm m} \\ C_{16} & = M_{\rm u} + M_{\rm m} \\ C_{17} & = C_{5} M_{\rm u} + C_{6} M_{\rm m} \\ C_{18} & = C_{10} + C_{17} \\ C_{19} & = C_{10} + M_{\rm u} (C_{5} + Q) + M_{\rm m} (C_{6} + Q) \\ C_{20} & = C_{10} g + M_{\rm u} g(C_{5} + Q) + M_{\rm m} (C_{6} + Q) \\ L_{\rm u} & = C_{5} + Q \\ L_{\rm m} & = C_{6} + Q \\ J_{\rm m} & = M_{\rm m} (Q^{2} + (C_{4} - 2L_{\rm g} )Q + L_{\rm g}^{2} - 2L_{\rm g} C_{6} + C_{3} ) \\ J_{\rm u} & = M_{\rm u} (Q^{2} + (C_{2} - 2L_{\rm g} )Q + L_{\rm g}^{2} - 2L_{\rm g} C_{5} + C_{1} ) \\ \end{aligned} $$

### Appendix 3: Double IP constants

$$ \begin{aligned} C_{1} & = 0.125R_{\rm w} \left( {M_{\rm m} + M_{1} + M_{{2{\rm l}}} + M_{\rm a} + M_{{2{\rm u}}} + M} \right) \\ C_{2} & = 0.5\left( {M_{\rm m} L_{1}^{2} + M_{1} L_{c1}^{2} + M_{{2{\rm l}}} L_{1}^{2} + M_{\rm a} L_{1}^{2} + M_{{2{\rm u}}} L_{1}^{2} + ML_{1}^{2} } \right) \\ C_{3} & = M_{{2{\rm l}}} L_{{{\rm c}2}}^{2} + M_{\rm a} L_{\rm a}^{2} \\ C_{4} & = 0.5M_{\rm m} \left( {\cos \alpha + \sin \alpha } \right) + 0.5M_{1} \left( {\cos \alpha + \sin \alpha } \right) \\ C_{5} & = M_{{2{\rm l}}} L_{1} L_{{{\rm c}2}} + M_{\rm a} L_{1} L_{\rm a} \\ C_{6} & = 0.5R_{\rm w} \left( {M_{{2{\rm l}}} L_{1} + M_{\rm a} L_{1} + M_{{2{\rm u}}} L_{1} + ML_{1} } \right) \\ C_{7} & = R_{\rm w} \left( {M_{{2{\rm l}}} L_{c1} + M_{\rm a} L_{\rm a} } \right) \\ C_{8} & = 2L_{{{\rm c}2}} + L_{{2{\rm u}}} \\ C_{9} & = 2L_{{{\rm c}2}} + 2L_{{2{\rm u}}} \\ C_{10} & = 4L_{{{\rm c}2}}^{2} + L_{{2{\rm u}}}^{2} + 4L_{{2{\rm u}}} L_{{{\rm c}2}} \\ C_{11} & = 4L_{{{\rm c}2}}^{2} + 4L_{{2{\rm u}}}^{2} + 8L_{{2{\rm u}}} L_{{{\rm c}2}} \\ C_{12} & = M_{\rm w} R_{\rm w}^{2} + J_{\rm w} \\ C_{13} & = 2J_{\rm w} + J_{IB} \\ C_{14} & = M_{1} L_{c1} + M_{\rm m} L_{1} + M_{{2{\rm l}}} L_{1} + M_{\rm a} L_{1} + M_{{2{\rm u}}} L_{1} + ML_{1} \\ C_{15} & = M_{{2{\rm l}}} L_{{{\rm c}2}} + M_{\rm a} L_{\rm a} \\ C_{16} & = M_{1} + M_{\rm m} + M_{{2{\rm l}}} + M_{\rm a} + M_{{2{\rm u}}} + M \\ C_{17} & = C_{15} + M_{{2{\rm u}}} C_{8} + MC_{9} \\ C_{18} & = C_{8} + C_{9} \\ C_{19} & = M_{{2{\rm u}}} + M \\ C_{20} & = 2C_{8} M_{{2{\rm u}}} + 2C_{9} M \\ C_{21} & = C_{3} + M_{{2{\rm u}}} C_{10} + MC_{11} \\ C_{22} & = 2M_{{2{\rm u}}} C_{8} + 2MC_{9} \\ C_{23} & = M_{{2{\rm u}}} L_{1} + ML_{1} \\ C_{24} & = C_{5} + M_{{2{\rm u}}} L_{1} C_{8} + ML_{1} C_{9} \\ C_{25} & = M_{{2{\rm u}}} R_{\rm w} + MR_{\rm w} \\ C_{26} & = C_{7} + M_{{2{\rm u}}} R_{\rm w} C_{8} + MR_{\rm w} C_{9} \\ C_{27} & = C_{1} + C_{12} \\ C_{28} & = R_{\rm w} \left( {M_{{2{\rm u}}} + M} \right) \\ \end{aligned} $$
